# Identification of genes related to sexual differentiation and sterility in embryonic gonads of Mule ducks by transcriptome analysis

**DOI:** 10.3389/fgene.2022.1037810

**Published:** 2022-10-26

**Authors:** Yu Yang, Xuelian Li, Shengqiang Ye, Xing Chen, Lixia Wang, Yunguo Qian, Qingwu Xin, Li Li, Ping Gong

**Affiliations:** ^1^ Institute of Animal Husbandry and Veterinary Science, Wuhan Academy of Agricultural Science, Wuhan, China; ^2^ Key Laboratory of Agricultural Animal Genetics, Breeding and Reproduction of Ministry of Education, Huazhong Agricultural University, Wuhan, China; ^3^ Institute of Animal Husbandry and Veterinary Medicine, Fujian Academy of Agricultural Sciences, Fuzhou, China

**Keywords:** transcriptome, sex, sterility, embryonic gonad development, gene, Mule ducks

## Abstract

The key genes of avian gonadal development are of great significance for sex determination. Transcriptome sequencing analysis of Mule duck gonad as potential sterile model is expected to screen candidate genes related to avian gonad development. In this study, the embryonic gonadal tissues of Mule ducks, Jinding ducks, and Muscovy ducks were collected and identified. Six sample groups including female Mule duck (A), male Mule duck (B), female Jinding duck (C), male Jinding duck (D), female Muscovy duck (E), and male Muscovy duck (F) were subjected to RNA sequencing analysis. A total of 9,471 differential genes (DEGs) and 691 protein-protein interaction pairs were obtained. Totally, 12 genes (*Dmrt1, Amh, Sox9, Tex14, Trim71, Slc26a8, Spam1, Tdrp, Tsga10, Boc, Cxcl14,* and *Hsd17b3*) were identified to be specifically related to duck testicular development, and 11 genes (*Hsd17b1, Cyp19a1, Cyp17a1, Hhipl2, Tdrp, Uts2r, Cdon, Axin2, Nxph1, Brinp2,* and *Brinp3*) were specifically related to duck ovarian development. Seven genes (*Stra8, Dmc1, Terb1, Tex14, Tsga10, Spam1,* and *Plcd4*) were screened to be specifically involved in the female sterility of Mule ducks; eight genes (*Gtsf1, Nalcn, Tat, Slc26a8, Kmo, Plcd4, Aldh4a1,* and *Hgd*) were specifically involved in male sterility; and five genes (*Terb1, Stra8, Tex14 Tsga10* and *Spam1*) were involved in both female and male sterility. This study provides an insight into the differential development between male and female gonads of ducks and the sterility mechanism of Mule ducks through function, pathway, and protein interaction analyses. Our findings provide theoretical basis for the further research on sex determination and differentiation of birds and the sterility of Mule ducks.

## Introduction

Avian gonad development can be divided into three stages. The first stage refers to the early embryonic stage when the genital ridges exhibiting “bipotential” trait form, and the sex-determining switch genes drive genital ridges to develop towards ovaries or testes. The second stage is the early and middle embryonic stage when the genital ridges further develop to form the testis or ovary, accompanied with the formation of a regulatory network composed of abundant genes to induce the sex differentiation of specific cells in the testis or ovary. The third stage refers to the one when male and female characteristics are maintained by hormones, and in this stage a series of genes are involved in the regulation of sexual characteristics maintenance (Barske and Capel 2010; Guioli, et al., 2020). Several critical genes reported to play important roles in gonadal development in mammals may also act in chick gonadal development (Oréal, et al., 2002; Feng, et al., 2007). For example, the SRY-like HMG box gene 9 (*Sox9*) is marker gene of testicular development, and cytochrome P450 family 19 subfamily A member 1(*Cyp19a1*) is ovarian developmental marker genes. In mammals, the expression of *Sox9* is activated by *Sry*, whereas the expression of *Sox9* must be initiated by another male-specific gene (probably a Z-linked gene) due to the lack of *Sry* in birds. Aromatase encoded by *Cyp19a1* is a key enzyme in the estrogen synthesis pathway (Elbrecht and Smith 1992), and aromatase is associated with the production of cortical layer and proliferation of germ cells and Sertoli cells. The expression pattern and expression stage of other sex-related genes such as Anti-Müllerian hormone gene (*Amh*), Steroidogenic factor-1 (*Sf1*), Wilm’s tumor1 gene (*Wt1*), and Gata binding protein 4 (*Gata4*) in birds were different from those in other species (Nachtigal, et al., 1998). The *Pitx2* gene related to asymmetric organ development has been reported to regulate the development of asymmetric gonad in chickens, and translocation experiments have revealed that this gene causes the degeneration of the right ovary in hens (Guioli and Lovell-Badge 2007). Although these genes mentioned above play a certain role in avian gonad development and participate in some developmental regulatory networks, they are not sex-determining genes. Therefore, it is necessary to screen related genes so as to gain insights into avian gonadal development.

Hybridization between distant genera can combine biological characteristics of distant species and break species constraints, thus driving genome evolution and new species formation (Grant et al., 2005; Mallet 2005; Mallet 2007). Nevertheless, there often exists reproductive isolation for evolutionarily well-differentiated taxa, therefore resulting in sterility of hybrids (Wünsch and Pfennig 2013). Chickens and quails belong to the genus *Gallus* and *Coturnix*, respectively, both of which belong to the Phasianidae of Galliformes of Aves. They have the same number of chromosomes (2n = 78) and their chromosome structures are highly conserved (Shibusawa, et al., 2001). The hybrid between chicken and quail belongs to intergeneric cross, and the sex differentiation is male-oriented from the 10th day of incubation. All the females die in the embryonic period and the hatched hybrids are only male with sterility trait (Ishishita, et al., 2016). Mule ducks are the offspring of intergeneric hybridization between *Cairina moschata* and *Anas platyrhynchos* domestica, and are also the products of distant hybridization. The comparison of the morphology of testicular germ cells between domestic ducks and Mule ducks showed that spermatogonia and primary spermatocytes were observed in seminiferous tubules of 52-week-old Mule ducks with secondary spermatocytes, spermatocytes, and sperms undetected. In contrast, the testes of all 9-week-old male domestic ducks contained sperms, indicating that meiotic failure is one of the reasons for Mule ducks sterility (Huang and Sung 1988). Additionally, the comparison the testicular morphology between Mule ducks and Peking ducks showed that the spermatogonia and spermatocytes of Mule ducks are disorderly arranged with no mature sperms found in the testis (Li, et al., 2020). Although infertility phenotypes have been observed in adult Mule duck, the genes responsible for sterility in Mule ducks remain largely unclear. By comparing the morphological characteristics of the 1/4, 1/2, and 3/4 of embryonic stage of the Mule ducks with those of their parents, our previous study (unpublished) found that male and female individuals formed the seminiferous tubules of the testis and the cortex and medulla of the ovary at the 1/2 embryonic stage, respectively. Especially in this stage (1/2 stage), the expression level of *Dmrt1* was highest, which was higher in males than in females, suggesting that the 1/2 embryonic stage might be a critical period for the development of gonads in Mule ducks. However, the underlying factors affecting sterility in early gonadal development remains to be investigated. Transcriptome sequencing technology can quickly and comprehensively screen related genes in specific avian gonadal development stages based on information about transcripts. In this study, we investigated the genes related to the early-stage gonad development of the Mule ducks with Jinding ducks and Muscovy ducks as parents. We constructed transcriptome libraries of Mule ducks, Jinding ducks and Muscovy ducks during 1/2 embryonic period *via* high-throughput sequencing. Our study screened 32 candidate genes responsible for sterility and sexual differentiation in the early gonadal development of Mule ducks. Our findings lay a foundation for elucidating the mechanism underlying avian gonad development.

## Materials and methods

### Sex identification

PCR amplification was performed with gonadal tissue DNA as a template, and gene-specific PCR primers were designed according to the *Chd1* gene on the sex chromosomes. The sex could be identified according to the observed size of particular bands. Only one band with a size of about 467 bp was considered as male, and two bands with a size of 467 bp and 326 bp were identified as female (Supplementary Figure S1). PCR was conducted in a 10 μl system containing 5 μl of 2x M5 HiPer plus Taq HiFi PCR mix (TaKaRa) 0.2 μl of each forward and reverse primer (Supplementary Table S1, 10 μM), 1 μl of DNA (approximate concentration of 100 ng/μl), and 3.6 μl RNase-free ddH_2_O. PCR was performed with 35 cycles of denaturing at 98°C for 10 s, annealing at 51°C for 20 s, and extension at 72°C for 30 s. PCR products were analyzed by 1.5% agarose gel electrophoresis (120 V, 200 mA, 20 min).

### Experiment material

The 500 eggs of Jinding ducks (domestic ducks), Muscovy ducks and their hybridization offspring Mule ducks were collected, respectively. When eggs were hatched to the 1/2 embryonic stage, the gonadal tissues were collected to extract DNA for sex identification. After sex identification, 10–20 paired gonads were combined into one sample. Six groups were respectively marked as A, female Mule ducks; B, male Mule ducks; C, female Jinding ducks; D, male Jinding ducks; E, female Muscovy ducks; F, male Muscovy ducks and a total of 18 samples were constructed with three biological replicates per group. The sample collection was approved by the Ethics Committee of Wuhan Academy of Agricultural Science.

### Total RNA extraction

Total RNA was extracted from all tissues using TRIzol Reagent (Invitrogen, Carlsbad, CA, United States) according to the manufacturer’s instructions. The integrity of the obtained RNA was evaluated using an Agilent Bioanalyzer 2100 system (Agilent Technologies, CA, United States).

### Library preparation for RNA sequencing

After quality evaluation of total RNA, high-quality total RNA samples were purified to obtain mRNA ending with Poly-A using the oligo (dT) magnetic beads, followed by fragmentation (about 300 bp) and cDNA synthesis with RNA as template. The RNA library was prepared using a total amount of 1 μg RNA per sample. The sequencing library was generated using NEB Next UltraTM RNA Library prep Kit for Illumina (NEB, United States) according to the instruction manual. A total of 18 cDNA libraries were constructed and paired-end sequenced using Illumina high-throughput sequencing platforms.

### Bioinformatics analysis

The software Cutadapt was used to remove adapter sequences and low-quality nucleotides from the raw data to obtain clean data. After quality control, clean reads were aligned to the *Anas platyrhynchos* reference genome (ftp://ftp.ensembl.org/pub/release100/fasta/anas_platyrhynch-os/dna/) using HISAT2 software (http://ccb.jhu.edu/software/hisat2/index.shtml). The Read Count value aligned to each gene using HTSeq software was used as the original expression level of the gene. Expression levels were normalized using the FPKM method (Trapnell, et al., 2010). Principal component analysis (PCA) of samples based on gene expression levels was performed using DESeq package in R. Hierarchical clustering of genes and samples was performed using pheatmap package in R. The Euclidean distance was used for calculating distances and the complete linkage was used in clustering. Gene ontology (GO) and KEGG pathway enrichment analyses were conducted using Blast2GO (Götz, et al., 2008) and bi-directional best-hit (BBH) assignment method on KEGG Automatic Annotation Server (KAAS) (Moriya, et al., 2007). The hypergeometric test was performed followed by FDR correction and items with corrected *p*-values less than 0.05 were considered to be significant or enriched. The data background is genes in the whole genome. The protein-protein interaction (PPI) prediction of DEGs was based on the retrieved associations from STRING (Jensen, et al., 2009)**.** Interaction score greater than 0.95 were screened and drawn with Cytoscape (Koonin and Rogozin 2003)**.**

### Quantitative real-time PCR (qRT-PCR)

RNA was reverse transcribed into cDNAs using the PrimeScriptTM RT reagent kit with gDNA Eraser (TaKaRa, Japan) following the manufacturer’s recommended procedures, and the resultant cDNAs were stored at -20°C. The quantitative real-time PCR (qRT-PCR) was conducted on an CFX-96/384 (Bio-Red, Hercules, CA, United States) in a 10 μl system containing 5 μl of 2 × SYBR Green qPCR Master Mix (Bimake, United States), 0.2 μl of each forward and reverse primer (Supplementary Table S1) (10 μM), 1 μl of cDNA (approximate concentration of 100 ng/μl), and 3.6 μl RNase-free ddH_2_O. The qRT-PCR was performed with 40 cycles of denaturing at 95°C for 15 s, annealing at 51°C for 30 s, and extension at 72°C for 20 s. *Gapdh* was used as an internal control. The experiments were conducted with at least four biological replicates and at least three technical replicates per biological replicate. Primer specificity was estimated by melting curves, and qPCR data were analyzed using 2-ΔΔCt method (Livak and Schmittgen 2001).

## Result

### mRNA expression profiles of embryonic gonads of Mule ducks, jinding ducks, and muscovy ducks

A total of 18 sequencing libraries of six sample groups were constructed (A, female Mule ducks; B, male Mule ducks;C, female Jinding ducks; D, male Jinding ducks; E, female Muscovy ducks; F, male Muscovy ducks) with three biological replicates per group. A total of 798216036 clean reads were obtained after paired-end sequencing on the Illumina sequencing platform, and the reference genome alignment results showed that the unique alignment rate was above 87% (Supplementary Table S2). The raw reads of our transcriptome data have been deposited into the NCBI Short Read Archive (SRA, http://www.ncbi.nlm.nih.gov/sra/) under accession number PRJNA874907.

### Screening of differentially expressed gene (DEGs)

**FIGURE 1:**
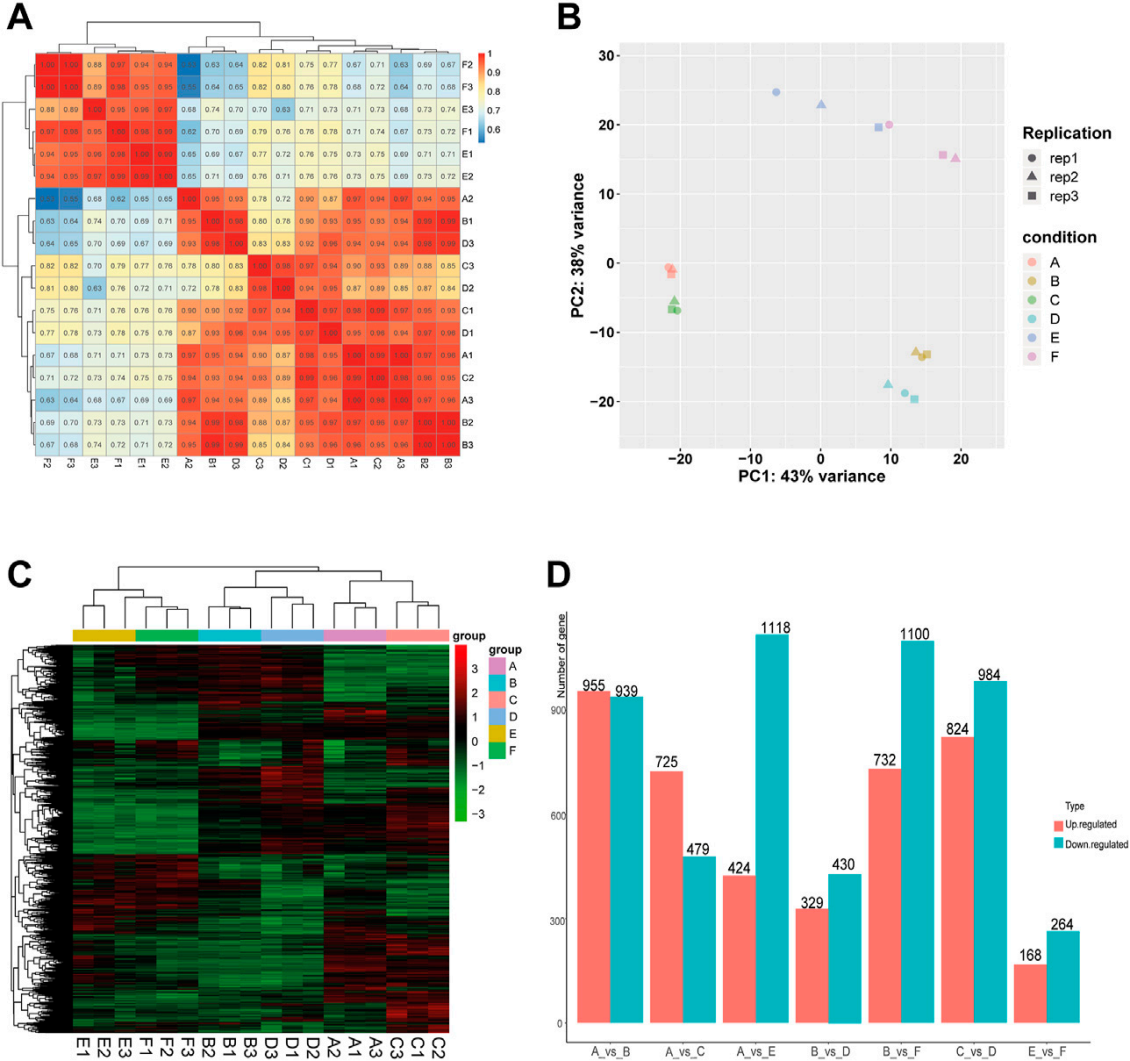
Correlation analysis of samples and screening of DEGs in the comparison between or within breeds (Mule ducks, Jinding ducks and Muscovy ducks). **(A)** Heatmap of correlations among groups. **(B)** Principal Component Analysis (PCA). Different shapes represent different replicates in the same group, and different colors represent different groups. **(C)** Hierarchical clustering analysis of DEGs. Columns indicate samples, and rows denote genes. The red represents the high gene expression and the green represents the low gene expression. **(D)** Statistics analysis of DEGs. The abscissa represents comparison groups, and the ordinate represents the number of DEGs. Red indicates up-regulated genes, and blue denotes down-regulated genes. A: female Mule ducks, B: male Mule ducks, C: female Jinding ducks, D: male Jinding ducks, E: female Muscovy ducks, F: male Muscovy ducks.

In order to evaluate the robustness of each group, we performed Spearman’s correlation analyses. The results showed that the average correlation coefficient of samples within the same group was above 0.9, and the average correlation coefficient between the Muscovy duck group and the other two groups was around 0.7 (Figure 1A). In addition, the principal component analysis (PCA) results showed a close distance within the same breed, while the samples from different breeds were more scattered (Figure 1B), and thus these samples were qualified for subsequent analysis.

To identify differentially expressed genes (DEGs), the hierarchical clustering analysis was performed with the threshold of *p*-value < 0.05 and absolute value of log2 FoldChange >1 (Figure 1C). A total of 9,471 DEGs were identified, including 1894 DEGs in the comparison of A vs. B, 1808 DEGs in C vs. D, 432 DEGs in E vs. F, 1542 DEGs in A vs. E, 1204 DEGs in A vs. C, 1832 DEGs in B vs. F, and 759 DEGs in B vs. D. The number of DEGs (432) identified in the comparison between male and female in Muscovy ducks was the smallest, while it was the largest between female and male in Mule ducks (1894). A total of 955, 732, and 725 up-regulated DEGs and 939, 1100, and 479 down-regulated DEGs were identified respectively in the comparison of male vs. female of Mule ducks, Muscovy ducks, Jinding ducks (Figure 1D). The volcano map of DEGs in each comparison was shown in Supplementary Figure.S2. As shown in the Veen diagram in Supplementary Figures S3, 131 shared genes were identified among A vs. B, C vs. D, and E vs. F, 139 shared genes were identified between A vs. C and A vs. E, 100 shared genes were identified between B vs. D and B vs. F.

### GO enrichment analysis

**FIGURE 2:**
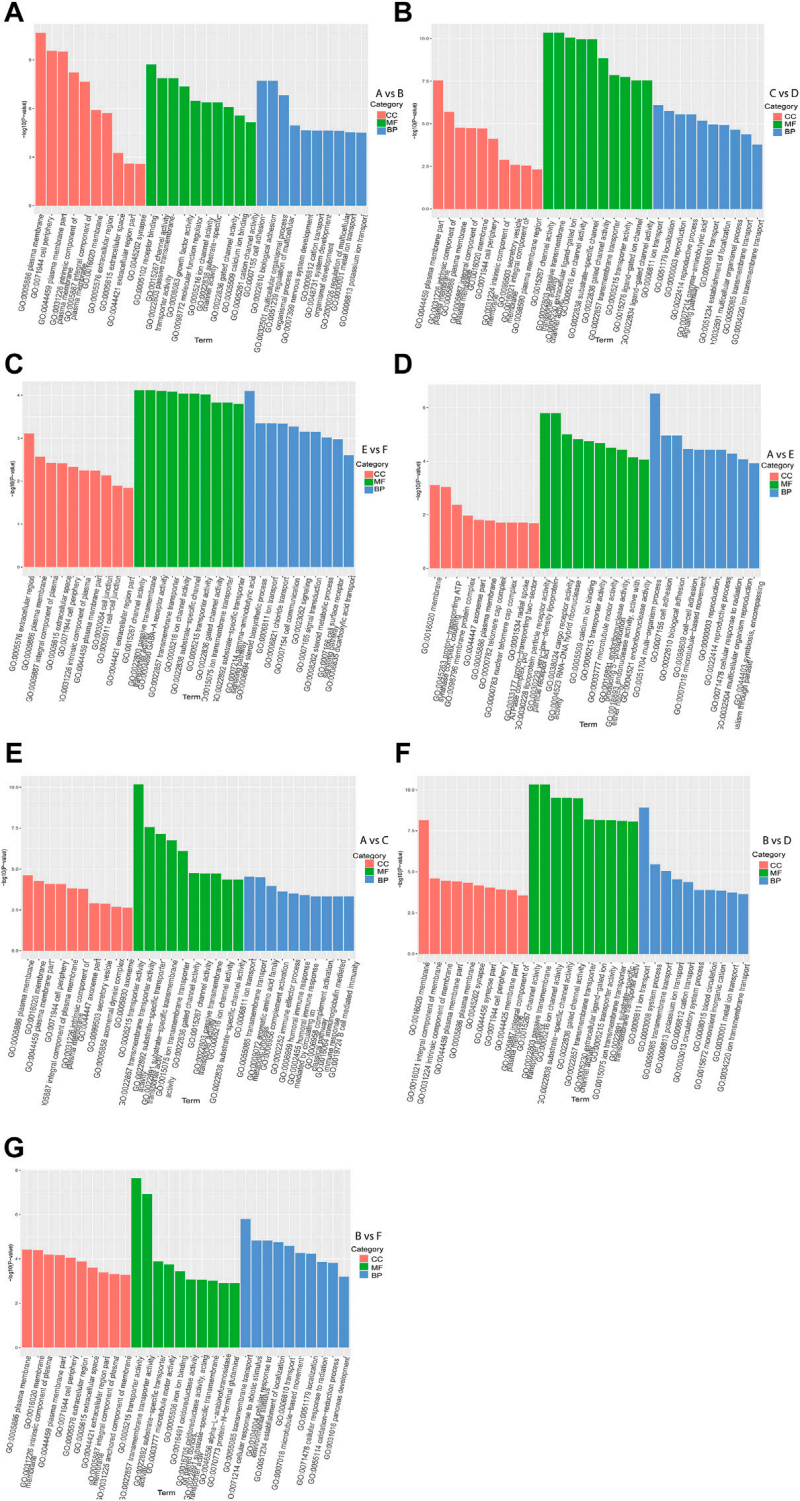
GO functional enrichment analysis of DEGs. The *x*-axis represents the secondary GO terms, the *y*-axis represents the -log10 *p*-value of enrichment of each term. Intra-breed comparisons between females and males include A vs. B. **(A)**, C vs. D. **(B)**, and E vs. F. **(C)**. Comparisons between Mule ducks and Muscovy ducks or Jinding ducks of same sex include A vs. E. **(D)**, A vs. C. **(E)**, B vs. D. **(F)**, and B vs. F. **(G)**. BP, biological processes; CC, cellular component; MF, molecular function.

GO enrichment analysis of DEGs in intra-breed comparison (A vs. B, C vs. D, and E vs. F) was performed. The top 10 shared GO terms in which DEGs were enriched mainly included ion transport or multicellular organismal process-related biological processes (BP), plasma membrane or extracellular space-related cellular component (CC), and ion channel activation related molecular function (MF) (Figures 2A–C). In A vs. B, of 64 DEGs involved in developmental process, 28 were up-regulated, 36 were down-regulated, of which 2 up-regulated DEGs (*Hsd17b3* and *Amh*) and one down-regulated DEG *Cyp17a1* were significantly enriched in sex differentiation. The GO enrichment analysis results also showed that three up-regulated DEGs (*Boc, Myocd*, and *Brinp1*) and three down-regulated DEGs (*Brinp3*, *Cdon*, and *Brinp2*) were significantly enriched in positive regulation of cell differentiation or developmental process. In C vs. D, one up-regulated DEG (*Spam1*) and four down-regulated DEGs (*Tsga10, Tdrp, Slc26a8, and Tex14*) were significantly enriched in developmental process and reproductive process. Additionally, there were four shared DEGs (*Axin2, Cxcl14, Uts2r,* and *Trim71*) among A vs. B, C vs. D, and E vs. F were significantly enriched in estrogen biosynthetic process, calcium ion transport, sex differentiation, and multicellular organism development.

In addition, in inter-breed female comparison (A vs. E and A vs. C), the top 10 shared GO terms in which DEGs were enriched mainly included immune response-or reproduction-related BP, telomere cap complex- or axonemal dynein complex-related CC, as well as ion channel activation- or receptor activation-related MF (Figures 2D,E). In A vs. E, a total of 7 DEGs were involved in reproductive process, of which *Tsga10, Stra8, Terb1, Spam1, Tex14* were up-regulated, and *Plcd4, Bmp15* were down-regulated. The down-regulated DEG *Plcd4* was also enriched in such biological processes as sexual reproduction and gamete generation. In A vs. C, four up-regulated DEGs (*Tra8, Dmc1, Terb1,* and *Tex14*) were significantly enriched in meiotic cell cycle. In inter-breed male comparison of B vs. D, three down-regulated DEGs (*Tsga10, Gtsf1,* and *Slc26a8*) were enriched in biological processes of male gamete generation and spermatogenesis, and in B vs. F, five up-regulated DEGs (*Tsga10, Stra8, Terb1, Plcz1,* and *Tex14*) and two down-regulated DEGs (*Plcd4* and *Hsd17b3*) were enriched in reproductive process (Figures 2F,G). Besides, four up-regulated DEGs (*Hgd, Tat, Kynu,* and *Kmo*) were involved in aromatic amino acid family catabolic process in B vs. F. It should be noticed that up-regulated DEG *Aldh4a1* was enriched in proline catabolic process.

### KEGG pathway analysis of DEGs

**FIGURE 3:**
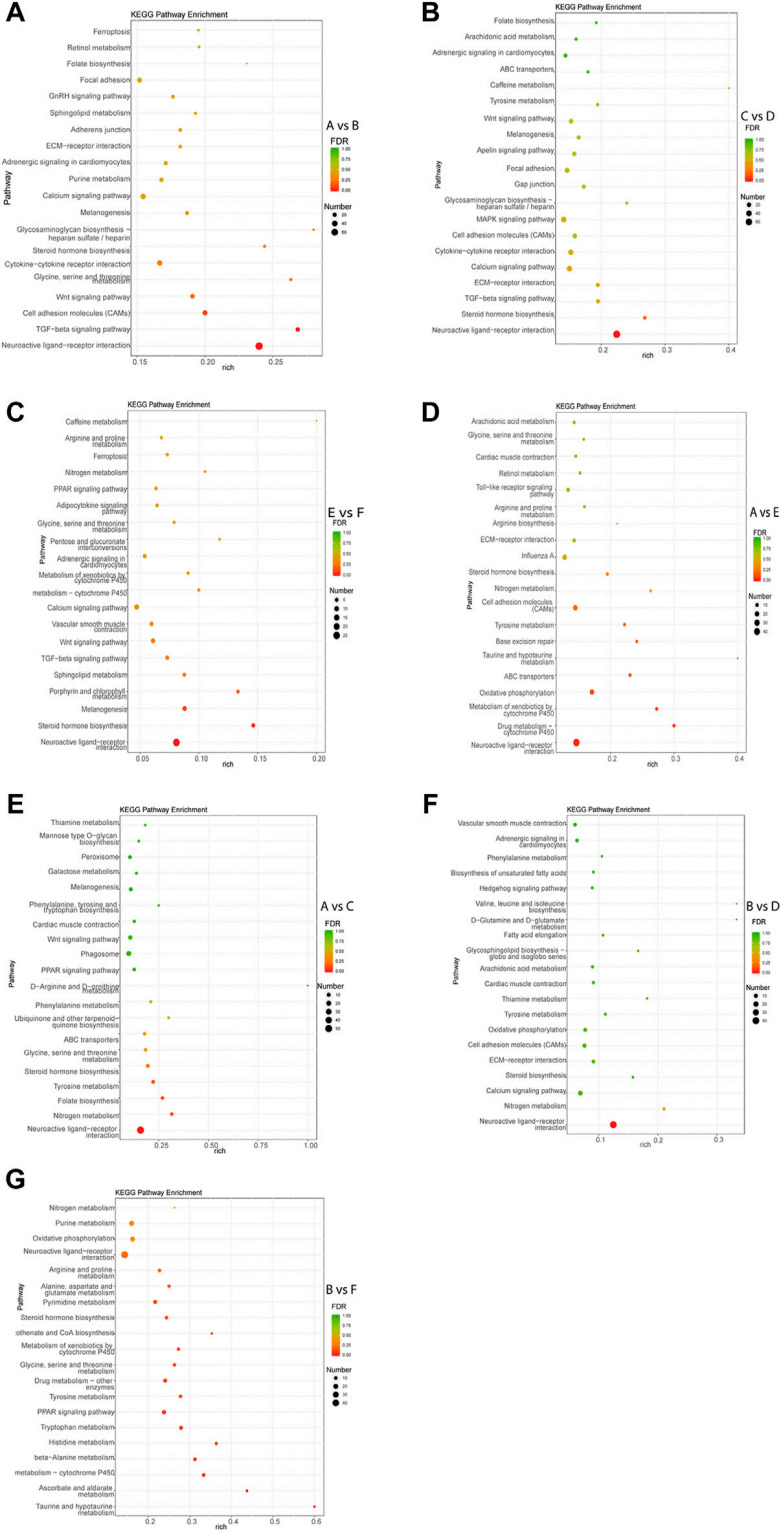
Top 20 pathways enriched with DEGs by KEGG pathway enrichment analysis. The Rich factor of *x*-axis is the ratio of the number of DEGs enriched in the pathway to the number of all annotated genes in the pathway. The size of the circle indicates the number of DEGs enriched in the pathway. The FDR (false discovery rate) indicates the enrichment degree of DEGs in a certain pathway. The closer to zero the FDR, the higher the enrichment degree. Intra-breed comparisons between females and males include A vs. B. **(A)**, C vs. D. **(B)**, and E vs. F. **(C)**. Comparisons between Mule ducks and Muscovy ducks or Jinding ducks of same sex include A vs. E. **(D)**, A vs. C. **(E)**, B vs. D. **(F)**, and B vs. F. **(G)**.

KEGG pathway analysis of each comparison group was performed. The results showed that the DEGs in intra-breed comparison were enriched in such signaling pathways as Calcium signaling pathway, Steroid hormone biosynthesis, and TGF-beta signaling pathway. Specifically, the DEGs in A vs. B, C vs. D, and E vs. F were also significantly enriched in GnRH signaling pathway, MAPK signaling pathway, Drug metabolism-cytochrome P450, respectively (Figures 3A–C). For inter-breed comparison, such signaling pathways as Drug metabolism-cytochrome P450 and Oxidative phosphorylation were significantly enriched with DEGs in A vs. E (Figure 3D). Signaling pathways such as Tyrosine metabolism and Steroid hormone biosynthesis were significantly enriched with DEGs in A vs. C (Figure 3E). Notably, most DEGs in B vs. D and B vs. F were enriched in amino acid metabolism-related pathways such as Tyrosine metabolism, beta-Alanine metabolism, Histidine metabolism pathways (Figures 3F,G). Furthermore, Calcium signaling pathway and Oxidative phosphorylation were significantly enriched with DEGs in B-vs-D.

### Protein interaction analysis

**FIGURE 4:**
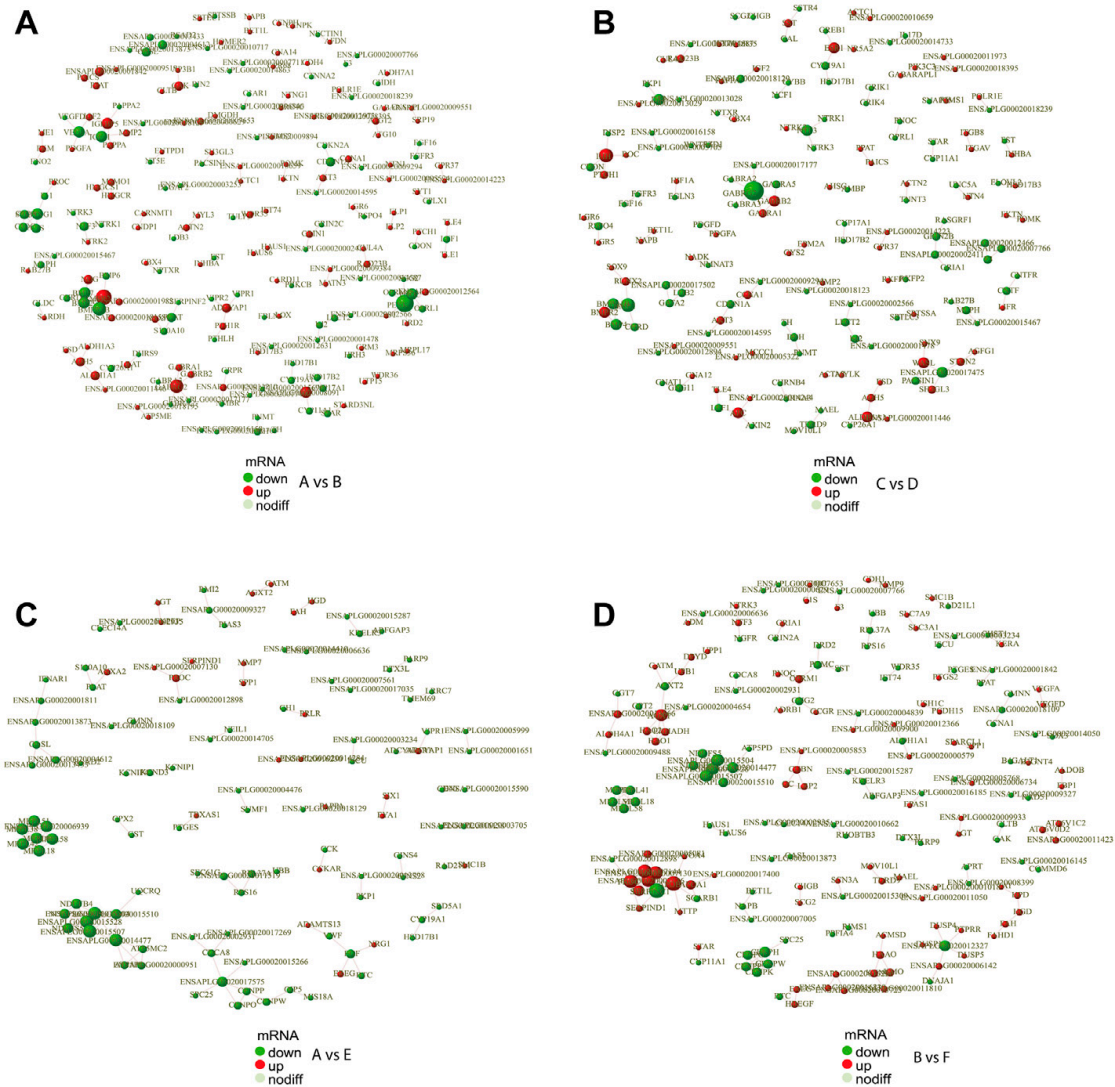
PPI network of DEGs. PPI network exhibits both protein interactions and expression levels. The dots indicate protein-encoding genes. Red indicates up-regulated genes, and green denotes down-regulated genes. PPI, protein-protein interaction; DEGs, differentially expressed genes. **(A)** PPI network of DEGs between female and male Mule ducks. **(B)** PPI network of DEGs between female and male Jinding ducks. **(C)** PPI network of DEGs between Mule duck and Muscovy duck females. **(D)** PPI network of DEGs between Mule duck and Muscovy duck males.

In order to reveal the relationship between genes, the DEGs were scanned by the STRING to construct the protein-protein interaction (PPI) network. The results showed that a total of 691 gene pairs were searched including 122 in A vs. E, 65 in A vs. C, 176 in B vs. F, 34 in B vs. D, 164 in A vs. B, 119 in C vs. D, and 11 in E vs. F. The PPI network contained 1893 DEGs and 164 (Score>0.95) interaction pairs in A vs. B (Figure 4A). Five genes in the subnetwork constructed by DEGs in A vs. B (HSD17B1, HSD17B2, CYP19A1, CYP17A1, and CYP11A1) were annotated to Steroid hormone biosynthesis. These five genes in the subnetwork in C vs. D were also annotated to Steroid hormone biosynthesis (Figure 4B). The interaction score of HSD17B1-CYP19A1 and CYP11A1-STAR was 0.981 and 0.986, respectively. Seven genes in another subnetwork in A vs. B (NOG, BMPR2, BMP6, BMP7, BMP2, BMPR1B, and CHRD) were annotated to TGF-beta signaling pathway (*p*-value = 7.7 E-5),and the interaction score of NOG-BMP7 was 0.989. In addition, 3 DEGs (CYP19A1, HSD17B1, and SRD5A1) in the subnetwork in A vs. E were annotated to the redox biological processes and lipid metabolism signaling pathway (Figure 4C). The resultant PPI network in B vs. F contained 1832 DEGs and 176 (Score>0.95) interaction pairs. And four genes (AGXT2, GPT2, ALDH4A1, and AGXT) in the subnetwork were annotated to amino acid metabolism-related pathways such as Alanine, aspartate, and glutamate metabolism pathways (Figure 4D). The PPI networks of DEGs in the comparisons of E vs. F, A vs. C, and B vs. D were shown in Supplementary Figure S4. And The protein interaction scores of some subnetworks were shown in Supplementary Table S3.

### Screening of key candidate genes related to gonadal development and sterility of Mule ducks

**FIGURE 5:**
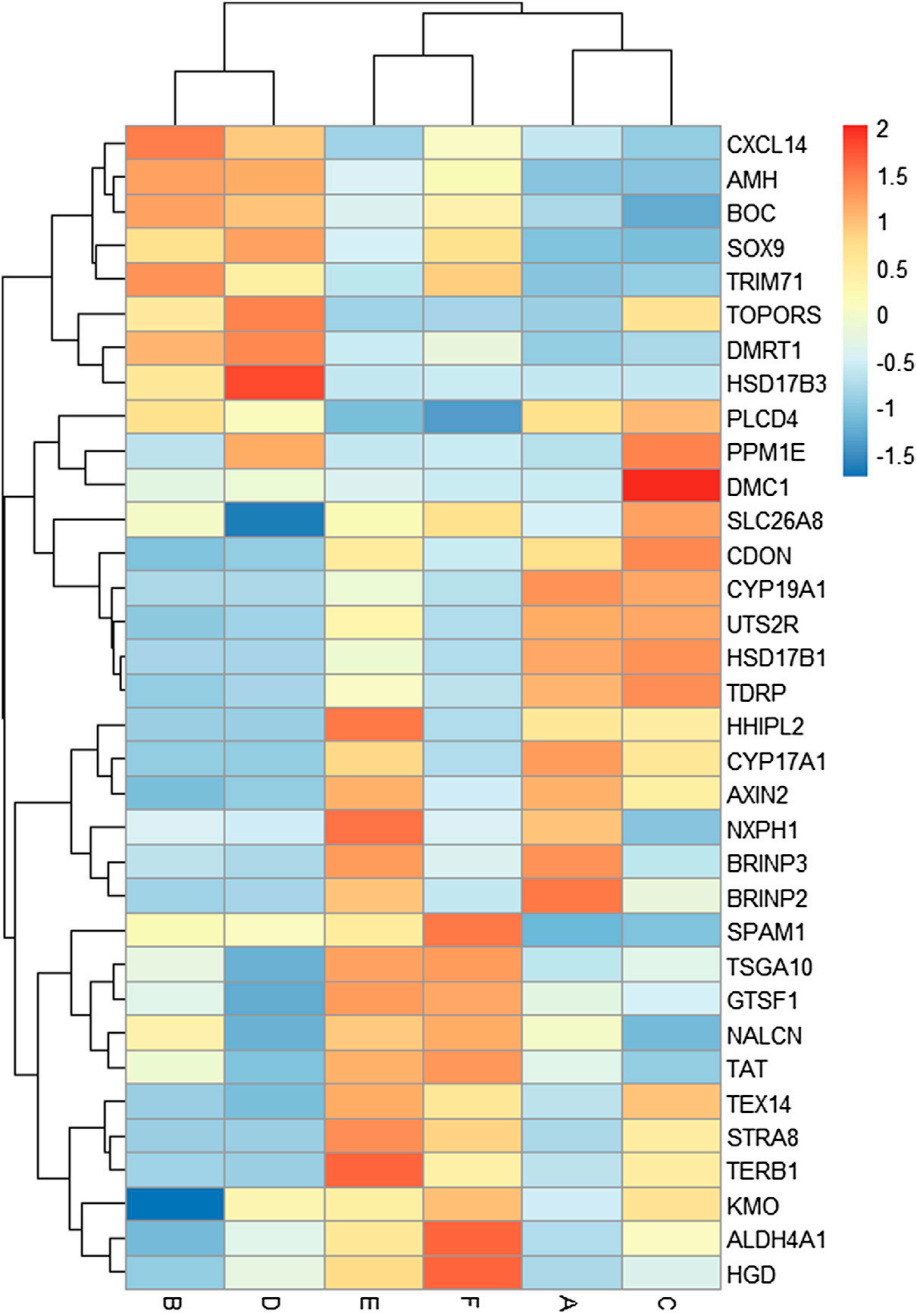
Heat map of the expression levels of key candidate genes related to gonadal development and sterility in Mule ducks. Red represents a high expression level, and blue indicates a low expression level. Columns and rows represent samples and genes, respectively. The color scale represents fold changes in gene expression.

A total of 34 key candidate genes were identified as gonadal development- or infertility-related genes according to our clustering analyses, functional enrichment analyses, and the published literature (Figure 5), of which 22 sex-biased DEGs (female-biased or male-biased), namely, *Dmrt1, Amh, Sox9, Tex14, Trim71, Slc26a8, Spam1, Tdrp, Tsga10, Boc, Cxcl14, Hsd17b3, Hsd17b1, Cyp19a1, Cyp17a1, Hhipl2, Uts2r, Cdon, Axin2, Nxph1, Brinp2,* and *Brinp3* were identified as sex-related genes in Mule ducks, Jinding ducks, and Muscovy ducks. Most of these 22 sex-related genes were annotated to sexual differentiation- and reproduction-related biological processes, of which, seven genes (*Cxcl14* *,* *Amh* *,* *Boc* *,* *Sox9* *,* *Trim71* *,* *Dmrt1, and Hsd17b3*) were strongly expressed in male gonads of at least two duck breeds and simultaneously lowly expressed in female gonads of all three duck breeds. The 11 genes (*Tdrp, Hsd17b1, Cyp19a1, Cyp17a1, Hhipl2, Uts2r, Cdon, Axin2, Nxph1, Brinp2,* and *Brinp3*) were highly expressed in female gonads of at least two duck breeds and meanwhile lowly expressed in male gonads of all three breeds. In addition, seven genes including *Stra8, Dmc1, Terb1, Tex14, Tsga10, Spam1,* and *Plcd4* were involved in meiotic cell cycle, reproductive process, and gamete generation, and they were identified as the genes causing female sterility in Mule ducks. The expression of three genes (*Stra8, Terb1*, and *Tex14*) in female gonads were lower in Mule ducks than in other two breeds. Thirteen genes including *Gtsf1, Nalcn, Tat, Slc26a8, Kmo, Plcd4, Aldh4a1, Hgd, Tsga10, Hsd17b3, Stra8, Terb1,* and *Tex14* involved in male gamete generation, spermatogenesis, sperm motility, proline catabolic process, and reproductive process were identified as the genes causing male sterility in Mule ducks. Among them, the expression of three genes (*Kmo, Aldh4a1,* and *Hgd*) in male gonads was lower in Mule ducks than in other two breeds. and the expression of five genes (*Tsga10, Gtsf1, Nalcn, Tat,* and *Slc26a8*) in male gonads of Mule ducks was lower than that of Muscovy ducks but higher than that of Jinding ducks.

### Verification of DEGs by qRT-PCR

**FIGURE 6:**
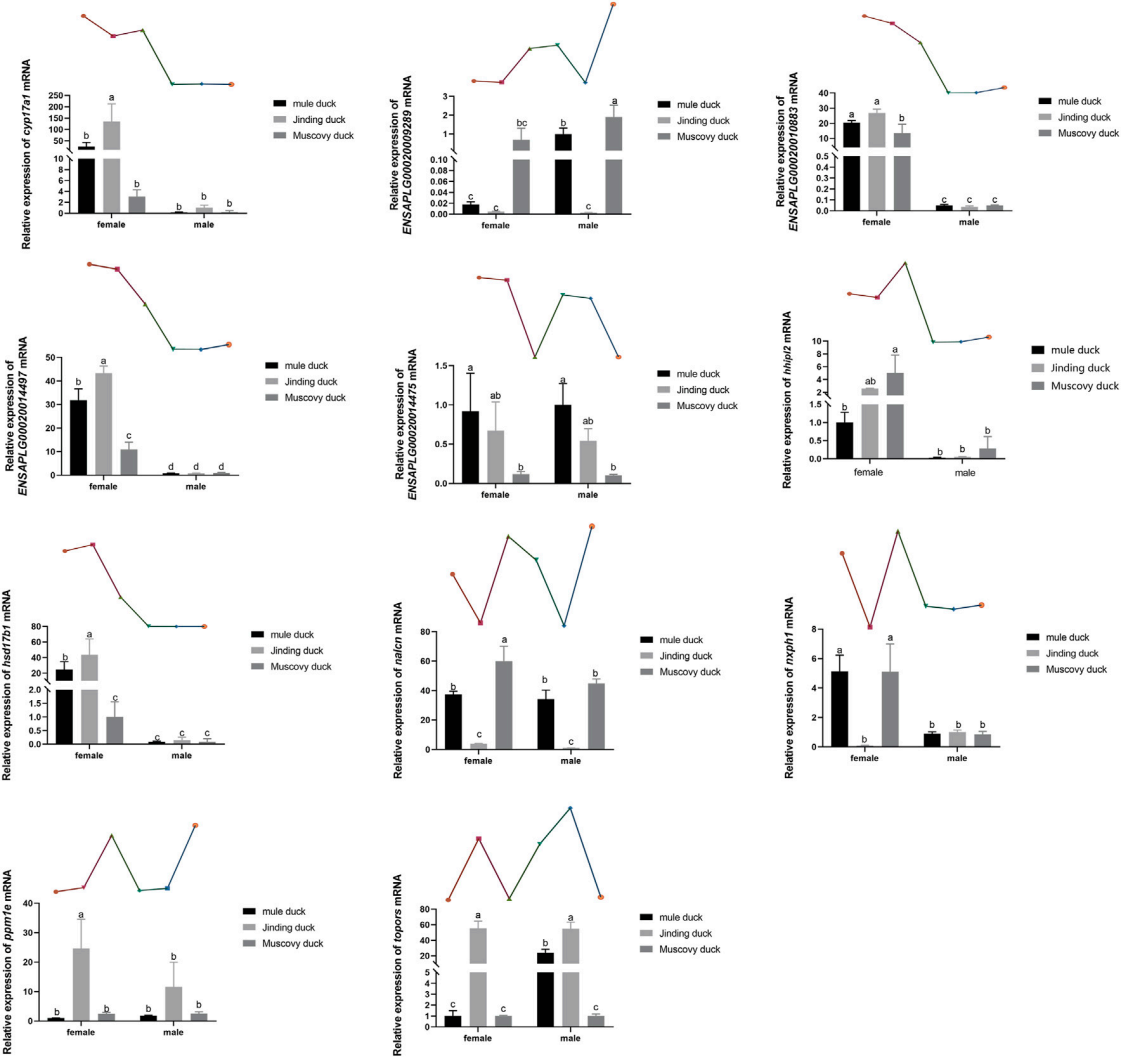
qRT-PCR validation of 11 DEGs identified by RNA-Seq. The gene expression level in the RNA-seq data indicated by line chart corresponds to that the qRT-PCR results displayed in bar chart. The data are expressed as mean ± standard deviation (SD). Each group has three biological replicates, and the lower-case letters above the bars represent significant differences.

Eleven DEGs were randomly selected for validation by qRT-PCR. As shown in Figure 6, six genes, namely, *Cyp17a1, Hhipl2, hsd17β1, Nxph1, ENSAPLG00020014497,* and *ENSAPLG0002-0010883*, were more highly expressed in males than in females of both three breeds. *ENSAPLG000200-14475* was more highly expressed in both females and males of Mule ducks than in those of Muscovy ducks. Particularly, the expression level of *topors* was higher in Mule duck females than males. The expression of *Nxph1* was higher in the females of Mule ducks and Muscovy ducks than their males. Eleven DEGs identified by qRT-PCR coincided with the RNA-Seq results, indicating the reliability of RNA-Seq results.

## Discussion

**FIGURE 7:**
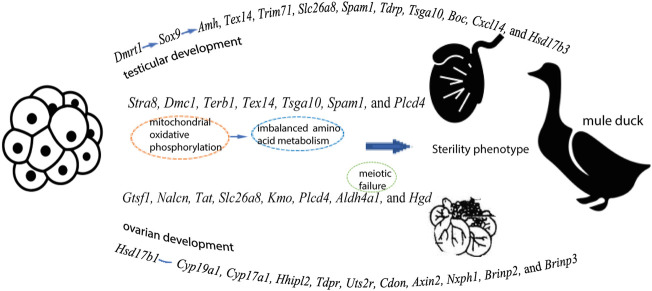
A speculation explaining the gene expression pattern in testes and ovaries, as well as the sterility in Mule ducks

From initiation of sex differentiation, sex-biased genes drive the gonad to develop toward the testis or ovary, which plays a key role in the formation and functions of testis or ovary. Z-linked *Dmrt1* has been reported to have important effect on gonadal development in multiple species (mammals, chicken, and fish) (Chen, et al., 2014). Our data showed that the expression levels of *Dmrt1, Amh, and Sox9* were higher in males than in females of all three duck breeds. The transcriptome analysis has revealed that *Amh* is significantly up-regulated after male differentiation by comparison before or after gonad differentiation in Muscovy ducks (Bai, et al., 2020). Moreover, the expression of *AmhR2* in male spermatogenic cords is disrupted after knockdown of *Dmrt1* during sexual differentiation in birds (Cutting, et al., 2014), implying that *Dmrt1* acts upstream of *Amh* to induce male initial differentiation and continues to play roles after differentiation. Additionally, the expression of *Amh* is regulated by the testicular factor *Sox9* (Arango, et al., 1999), and *Dmrt1* has been reported to possibly bind to the enhancer of *Sox9* to regulate sex differentiation (Primakoff and Myles 2002). Based on these findings, it can be speculated that *Dmrt1* might regulate *Sox9*, thus controlling the expression of *Amh* during sex differentiation of birds, and thus gene regulation plays an important role in the process of gonadal differentiation and development (Figure 7).

Spermatogenesis can be divided into three principal stages: mitotic proliferation of spermatogonia, genome reduction of meiotic spermatocytes, and morphological transformation from haploid spermatids to sperm. Incomplete cytokinesis makes daughters connected by stable intercellular bridge to form germline cysts, thus sharing gene products (Braun, et al., 1989; Greenbaum, et al., 2011). Testis-expressed 14 (*Tex14*) is a novel protein localized to intercellular bridges of germ cells (Greenbaum, et al., 2006), and a study of adult mouse testis has shown that the *Tex14* protein is essential for blocking cytokinesis and converting transient midbody loops into stable intercellular bridges (Kim, et al., 2015). During spermatogenesis of *Tex14* ^*−/−*^ mice, although diploid spermatogonia are subjected to transport, proliferation, and early meiotic marker expression, these processes stopped before the first meiosis, suggesting *Tex14* mutation was likely to prevent the function of intercellular bridge, thereby leading to meiosis defects, eventually causing sterility (Greenbaum, et al., 2006). Our data showed that *Tex14* was highly expressed in male Mule ducks but lowly expressed in females. Besides, *Trim71* deletion leads to a reduction in the undifferentiated spermatogonia population, and blocks the differentiation of male reproductive system of mice (Du, et al., 2020). In this study, the expression of *Trim71* was higher in males of three breeds than in females. Spam1, sperm membrane protein in mammals, is widely conserved across species (Lathrop, et al., 1990) and it is expressed in both testis and epididymis (Zheng, et al., 2001; Zhang, et al., 2004). As a multifunctional protein, Spam1 with multiple domains plays multiple roles in fertilization. Testis development-related protein (*Tdrp*) is a nuclear factor, and in *Tdrp*-deletion mouse model, many sperm motility indices such as average path velocity (VAP), straight line velocity (VSL), and curvilinear velocity (VCL) are significantly lowered (*p* < 0.05) (Mao, et al., 2016), implying that deletion of *Tdrp* impairs sperm motility. In our study, *Spam1, Tsga10, Tdrp, Slc26a8*, and *Tex14* were linked to sexual reproduction and reproductive process. *Boc* and *Cxcl14* displayed a high expression in males but a low expression in females in all three breeds, and our GO analysis showed that these two genes were involved in positive regulation of cell differentiation or developmental process.

The final step in androgen biosynthesis in the testis is the conversion of androstenedione (Δ4) into testosterone (T, Testosterone), and this step is catalyzed by 17β-HSD-3 (Twesten, et al., 2000). The increasing evidence has revealed that 17β-HSD-3 is expressed in Leydig cells, whose mutations cause a rare 46, XY disorder of sex development (DSD) (Folsom, et al., 2019), and it was first reported in 1971 that the phenotype of 17β-HSD-3 deficiency was an autosomal recessive inheritance (Saez, et al., 1971; Saez, et al., 1972). Our data showed that *Hsd17b3* located on the Z chromosome of ducks was more highly expressed in males than in females in Mule ducks and Jinding ducks. Besides, *Hsd17b3* was significantly enriched in sex differentiation GO term. In contrast, *Hsd17b1* was more highly expressed in females than in males in all three breeds, which was consistent with the observation during the gonad developmental stage in flatfish (Zou, et al., 2020). Aromatase encoded by *Cyp19a1* can convert androstenedione and testosterone into estrone (E1) and estradiol (E2), respectively. *Hsd17b1* can efficiently catalyze the conversion of E1 with low biological activity into E2 with high biological activity (Day, et al., 2008). Overexpression of human-derived *Hsd17b1* leads to masculinization of internal and external genitalia in female mice (Saloniemi, et al., 2009). In this study, the interaction score between *Hsd17b1* and *Cyp19a1* was 0.981, and these two genes were annotated to the Steroid hormone biosynthesis signaling pathway. Cytochrome P450 family 17 subfamily A member 1 (*Cyp17a1*), as an important member of the cytochrome P450 subfamily, exhibits both hydroxylase and lyase activities, and it is expressed in the Leydig cells and the granulosa cells of ovarian follicles (Zhou, et al., 2007). Mutation of *Cyp17a1* results in female-to-male sex reversal, but sex reversal was successfully rescued by 17β-estradiol (E2) or testosterone (T) in XX Nile tilapia (Yang, et al., 2021). Consistent with the expression pattern of *Hsd17b1*, *Cyp17a1* was highly expressed in females but lowly expressed in males in all three duck breeds, and *Cyp17a1* was involved in sex differentiation, suggesting that *Hsd17b1* and *Cyp17a1* might be key candidate genes related to gonadal differentiation and development. Furthermore, five genes (*Hhipl2, Tdrp, Uts2r, Cdon,* and *Axin2*) exhibited high expression in females but low expression in males in all three duck breeds, of which *Uts2r* and *Cdon* were involved in multicellular organismal process. *Nxph1, Brinp2,* and *Brinp3* were highly expressed in females but lowly expressed in males in Mule ducks and Muscovy ducks. of which *Brinp2* and *Brinp3* were involved in regulation of developmental process.

Only after meiosis can diploid progenitor cells produce haploid gametes (oocytes and sperm). In mammals, meiosis is initiated in different life stages for different sexes, and female germ cells enter meiosis shortly after sex determination during embryogenesis, while male germ cells do not enter meiosis until puberty (Goetz, et al., 1984; Spiller, et al., 2012). Our inter-breed female comparison of Mule ducks, Jinding ducks, and Muscovy ducks revealed that *Stra8, Dmc1, Terb1,* and *Tex14* were involved in meiotic cell cycle, of which 3 genes (*Stra8*, *Terb1,* and *Tex14*) were more highly expressed in Jinding duck females and Muscovy duck females than in Mule duck females. Stimulated by retinoic acid gene 8 (*Stra8*) is considered a key regulator of meiosis in mammals (Anderson, et al., 2008). In female mice, *Stra8* is expressed in embryonic ovarian germ cells before entering meiosis (Menke, et al., 2003). Although early mitosis of germ cells is normal in female embryos lacking *Stra8* gene function, these germ cells are unable to undergo DNA replication before entering meiosis and chromosome condensation, synapsis, and recombination processes during meiotic prophase, implying that initiation of the meiotic program is dependent on the function of *Stra8* in female embryonic germ cells (Baltus, et al., 2006). DNA meiotic recombinase 1 (*Dmc1*), a meiosis-specific gene, is crucial for assembling DNA double strand breaks (DSBs) site in homologous recombination and for searching DNA sequences located on homologous chromatids (Cao, et al., 2021). *Dmc1-*knockout mice have no follicles and a smaller ovary (Biswas, et al., 2021). Follicular development and spermatogenesis completely fail after *Dmc1* homozygote knockout in mice, and in humans, *Dmc1* is required for spermatogenesis but not for oogenesis (Cao, et al., 2021). In addition to the effect of *Spam1* on spermatogenesis mentioned above, the expression of *Spam1* in the reproductive tract of female mice was 3–10 times lower than that of males, and its expression in the uterus was unsteady during the estrous cycle (Zhang and Martin-DeLeon 2003). In this study, *Spam1* was expressed lower in female Mule ducks than in female Muscovy ducks, and it was involved in sexual reproduction, reproduction, and gamete generation.

Our inter-breed comparison of males (B vs. D and B vs. F) revealed that DEGs were significantly enriched in biological processes such as male gamete generation, spermatogenesis, proline catabolic process. Of these DEGs, the expression of four genes (*Gtsf1, Nalcn, Tat,* and *Slc26a8*) were higher in male Mule ducks than in male Jinding ducks but lower than in male Muscovy ducks. The three genes (*Kmo, Aldh4a1,* and *Hgd*) were lowly expressed in male Mule ducks whereas highly expressed in males of the other two breeds. These results suggested that the above-mentioned seven genes were related to duck male sterility. Gametocyte-specific factor 1 (*Gtsf1*) is evolutionarily highly conserved. Primary spermatocytes of *Gtsf1* ^*−/−*^ male mice stopped proliferation before the zygotene of the first meiotic prophase, thus causing massive germ cell apoptosis on postnatal day 14, eventually leading to male sterility (Yoshimura, et al., 2009). One previous study has shown that sperms with the phospholipase C delta 4 (*Plcd4*) gene homozygote deleted fail to initiate acrosome reaction, thereby resulting in male sterility or small number per litter in mice (Fukami, et al., 2001). DEGs in the comparison of B vs. D were involved in calcium signaling pathway in this study. Notably, the *Slc26a8* gene is expressed in the plasma membrane of male germ cells at the spermatocyte and sperm stages (Toure, et al., 2001). The deletion of *Slc26a8* results in the sperm capacitation impairment, flagellum structure defects, abnormal mitochondrial sheath assembly, and sperm motility reduction, thus causing sterility in male mice (Rode, et al., 2012). The *Kmo-*encoded enzyme operates at a key branch point in the kynurenine pathway, and the deletion of *Kmo* gene alters mitochondrial morphology and function (Maddison, et al., 2020). These previous findings indicate that sterility of Mule ducks might be related to mitochondria. Mitochondria are recognized as critical sites of energy conversion and metabolism in sperm, and they play critical roles in calcium homeostasis maintenance, spermatogenesis, apoptosis, and fertilization (Friedman and Nunnari 2014). Interestingly, mitochondria are identified as a major source of reactive oxygen species (ROS) in mammalian sperm (Koppers, et al., 2008). The oxidative phosphorylation (OXPHOS) in sperm mitochondria produces an appropriate amount of ROS, which is necessary for sperm tyrosine phosphorylation and cholesterol efflux, sperm maturation, capacitation, and sperm-oocyte binding (Samanta, et al., 2018). Approximately 30–40% of male infertility cases are associated with the elevated mitochondrial ROS levels (Jose-Miller, et al., 2007). The OXPHOS complex is present in the folded and curved part of the mitochondrial inner membrane. Pyruvate has been reported to be converted to acetyl-CoA to provide fuel for OXPHOS, and perturbation of OXPHOS makes spermatogenesis highly sensitive (Varuzhanyan and Chan 2020). In this study, DEGs in both B vs. D and B vs. F were enriched in oxidative phosphorylation pathway, particularly, enriched in the signaling pathway of pantothenate and CoA biosynthesis in B vs. F, implying that abnormal oxidative phosphorylation of mitochondria in germ cells might be responsible for the sterile phenotype of Mule ducks. As shown in Figure 3, many amino acid metabolism pathways were significantly enriched in the comparison of males across different breeds. *Aldh4a1* belongs to the aldehyde dehydrogenase superfamily, and it is associated with type II hyperprolinemia (HPII) (Geraghty, et al., 1998; van de Ven, et al., 2014), whose patients suffer from fibroblast mitochondrial dysfunction. *Aldh4a1* expression is significantly decreased in oocytes of postpartum mouse (Esteves, et al., 2011). Proline acts as a central amino acid in cellular redox control (Yen and Curran 2021). Antioxidant drugs can alleviate function impairment of sperm mitochondria induced by proline catabolism disorder (Yen and Curran 2021). More studies of amino acids have shown that homocysteine level is increased in sperm from patients with severe disruption of semen proteins (Kralikova, et al., 2017), that d-Aspartate (D-Asp) and N-methyl-d-aspartate (NMDA) promote spermatogenesis by stimulating the biosynthesis of sex steroid hormones (Di Fiore, et al., 2014), that D-Asp and NMDA can increase the expression of OXPHOS complex (Falvo, et al., 2022). Additionally, tyrosine phosphorylation is regulated by redox signaling (Chiarugi and Cirri 2003). Based on these findings, we speculated that in 1/2 embryonic stage of Mule ducks, inordinate amino acid metabolism caused by mitochondrial OXPHOS could be considered to be one of the reasons for sterile phenotype of Mule ducks (Figure 7).

Five genes (*Tsga10, Terb1, Stra8, Tex14,* and *Spam1*) were predicted to be associated with sterility in male and female Mule ducks. Our data showed that the expression level of these five genes was significantly lower in Mule ducks than that in Muscovy ducks for both males and females. In this study, *Tsga10* and *Spam1* were found to be potentially related to the female sterility in Mule ducks, of which testis-specific gene antigen 10 (*Tsga10*) has been reported to be highly expressed in various species, especially in the middle and main parts of sperm tails (Modarressi, et al., 2004). In *Tsga10* ^*+/−*^ mice generated by CRISPR/Cas9, the disordered mitochondrial sheath resulted in the reduced sperm motility (Luo, et al., 2021). It has been reported that *Stra8, Terb1*, and *Tex14* involved in meiosis of germ cells are also closely related to male sterility (Greenbaum, et al., 2006; Anderson, et al., 2008; Shibuya, et al., 2014). Our inter-breed female comparison showed that *Terb1, Stra8,* and *Tex14* were enriched in meiotic cell cycle. However, our inter-breed male comparison indicated that no genes were enriched in meiotic cell cycle, instead, some genes were enriched in mitochondrial oxidative phosphorylation and amino acid metabolism. Such difference might be due to the fact that male germ cells did not enter the meiotic process until puberty, but our samples were derived from the 1/2 embryonic stage (before meiosis). Furthermore, the imbalance between OXPHOS and spermatogonia glycolysis could lead to meiosis failure (Li Y, et al., 2021), which suggested that amino acid metabolism disturbance induced by mitochondrial oxidative phosphorylation might be reason for meiosis failure in male Mule ducks, further resulting in male Mule duck sterility (Figure 7). It should be noted that merely a few amino acid metabolism pathways were enriched by DEGs in inter-breed female comparison in this study (Figure 3). One previous study has shown that the maintenance of redox homeostasis can promote meiotic maturation of porcine oocytes (Li Z, et al., 2021). These findings jointly suggested that amino acid metabolism imbalance induced by mitochondrial oxidative phosphorylation might be one of the early causes behind meiotic failure, which was consistent with our speculation on the reason for male Mule duck sterility.

## Conclusion

Taken together, the transcriptome analysis of 1/2 embryonic gonads of three duck breeds was performed by RNA-seq. A total of 9471 DEGs were identified by intra- and inter-breed comparison between males and females of Mule ducks, Muscovy ducks, and Jinding ducks. Twelve genes (*Dmrt1, Amh, Sox9, Tex14, Trim71, Slc26a8, Spam1, Tdrp, Tsga10, Boc, Cxcl14,* and *Hsd17b3*) were identified to be specifically related to testicular development of ducks, and 11 genes (*Hsd17b1, Cyp19a1, Cyp17a1, Hhipl2, Tdrp, Uts2r, Cdon, Axin2, Nxph1, Brinp2,* and *Brinp3*) specifically related to the female. Seven genes (*Stra8, Dmc1, Terb1, Tex14, Tsga10, Spam1,* and *Plcd4*) were screened to be specifically responsible for the female sterility of Mule ducks, eight genes (*Gtsf1, Nalcn, Tat, Slc26a8, Kmo, Plcd4, Aldh4a1* and *Hgd*) for male sterility, and five genes (*Terb1, Stra8, Tex14 Tsga10* and *Spam1*) for both female and male sterility. This study lays a solid foundation for elucidating the mechanism of avian gonad development.

## Data Availability

The datasets presented in this study can be found in online repositories. The names of the repository/repositories and accession number(s) can be found in the article/Supplementary Material.
