# Supplementary figures and images for: Identification of genes related to sexual differentiation and sterility in embryonic gonads of Mule ducks by transcriptome analysis
Source: Front Genet. 2022 Oct 26;13:1037810. doi: 10.3389/fgene.2022.1037810 (PMC9643717; doi:10.3389/fgene.2022.1037810)

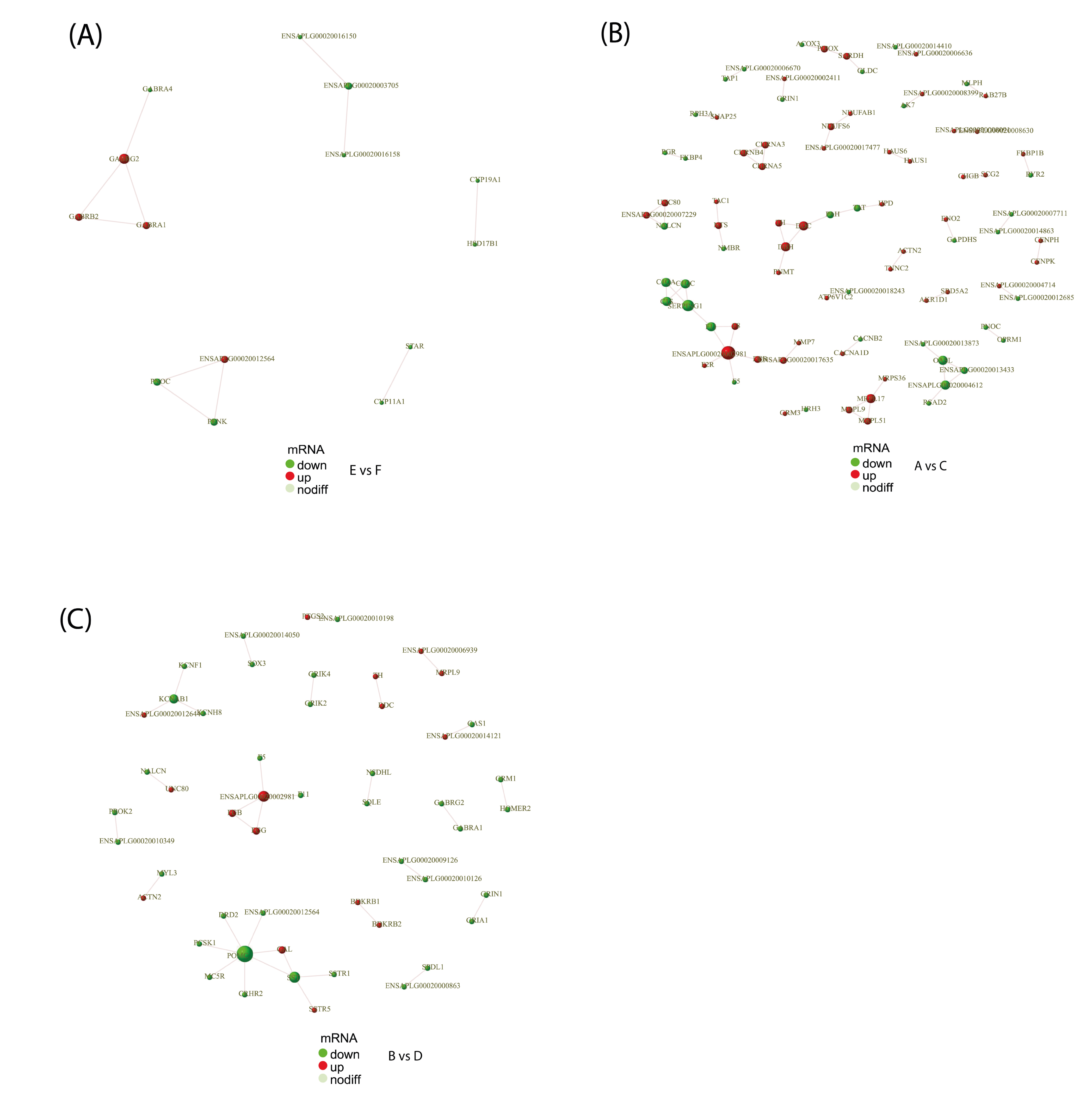

Supplement: Supplementary file 1 [file Image4.TIF]

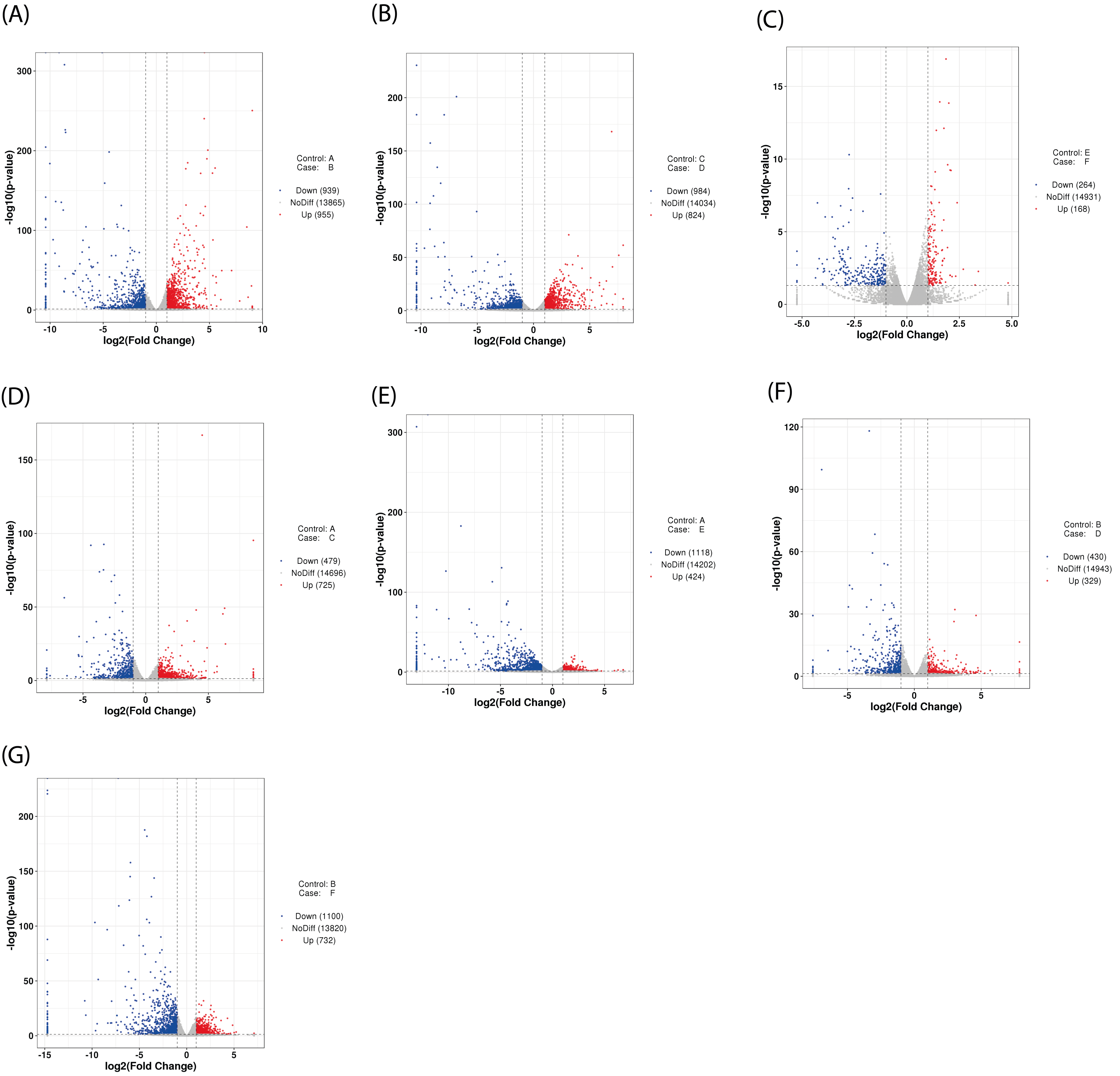

Supplement: Supplementary file 2 [file Image2.TIF]

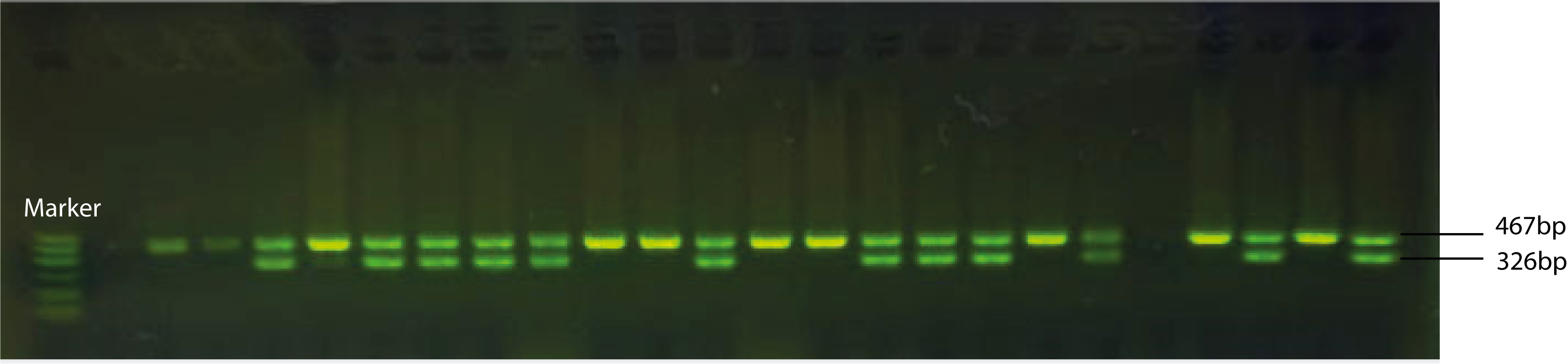

Supplement: Supplementary file 3 [file Image1.TIF]

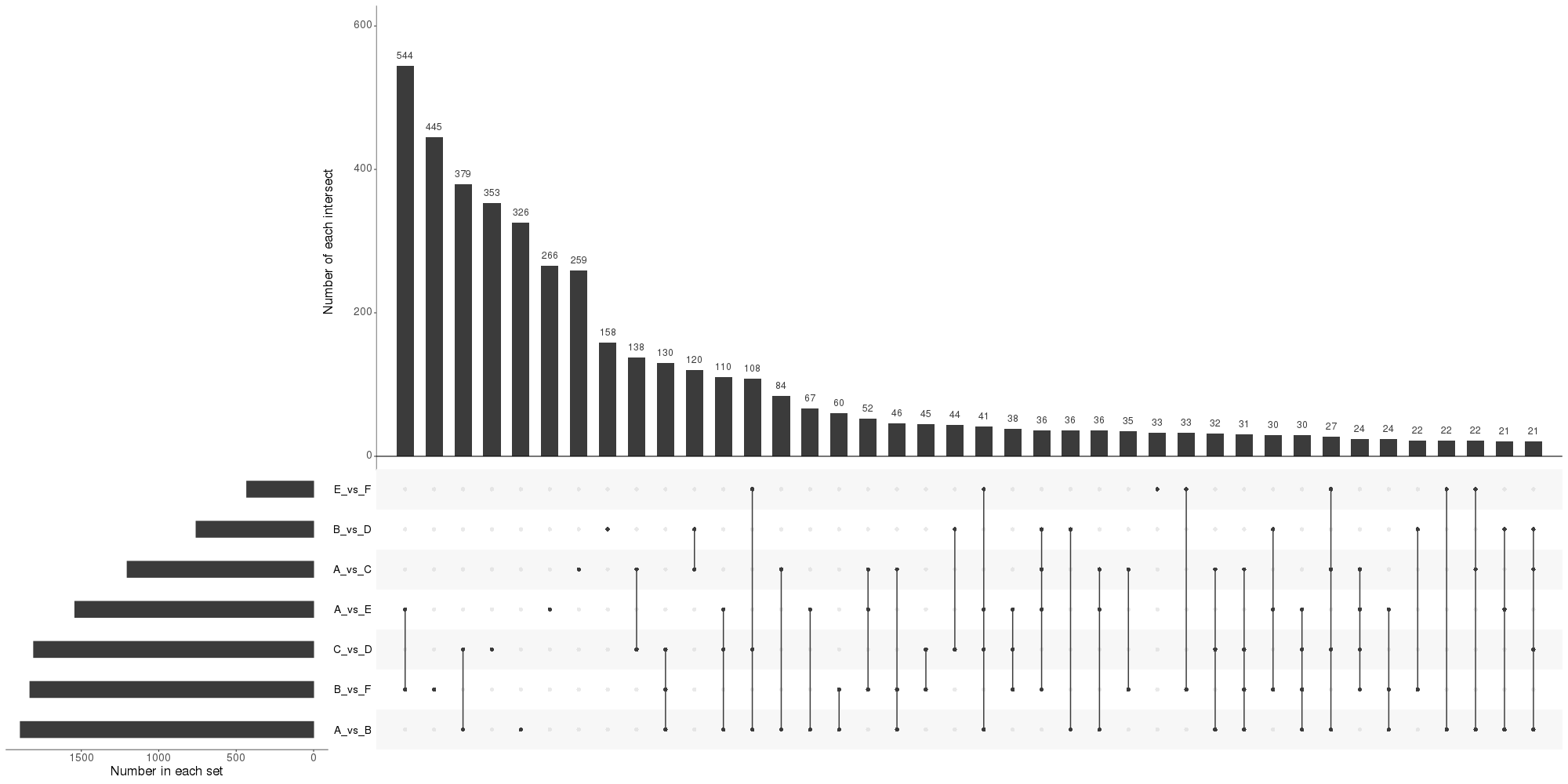

Supplement: Supplementary file 4 [file Image3.PNG]
