# Supplementary material for: Identification of genes related to sexual differentiation and sterility in embryonic gonads of Mule ducks by transcriptome analysis
Source: Front Genet. 2022 Oct 26;13:1037810. doi: 10.3389/fgene.2022.1037810 (PMC9643717; doi:10.3389/fgene.2022.1037810)
Supplement: Supplementary file 5 [file DataSheet1.docx]

**Table S1** **Primers used for qRT-PCR and PCR**

| **Primer name** | **Primer sequence（5’ - 3’）** | **Production length** |
| --- | --- | --- |
| *chd1-F*  *chd1-R*  *cyp17a1-F*  *cyp17a1-R*  *topors-F* | TGCAGAAGCAATATTACAAGT  AATTCATTATCATCTGGTGG  CCTGCTCCACTACCCTGA  TTGTCCCACTCCTTCTCG  TGGGAGACAGGCGTGCT | 273bp  189bp |
| *topors-R*  *hsd17b1-F*  *hsd17b1-R*  *nxph1-F*  *nxph1-R*  *hhipl2-F*  *hhipl2-R*  *ppm1e-F*  *ppm1e-R*  *nalcn-F*  *nalcn-R*  *ENSAPLG00020014475-F*  *ENSAPLG00020014475-R*  *ENSAPLG00020014497-F*  *ENSAPLG00020014497-R*  *ENSAPLG00020009289-F*  *ENSAPLG00020009289-R*  *ENSAPLG00020010883-F*  *ENSAPLG00020010883-R* | AAGCAGCGGTCCAGGTAG  CAGGTTCAAAGTGTTCGC  CCCCGAAGAGATTCACCT  AGTTTGAGCGGCGAGGAT  GTCGGCTGATGGACAAGT  GAATTGAAGGTTTTGGCC  TCAGGAGGGATGCGATAA  TTCAACCTTGAGGACCAA  TTCCACAACGCAAACTCT  GAACGGCTGCATAATGGT  AAATCGGCTCAACTCCTG  TGTCCTTGGCTTACCTGC  GCTTAGGAGGAGGACGAA  CTACAAAGGTATCCGTCCAG  CACCACGATTCAGCAACT  TGGGCAGTGATGTGTTG  GCCAAATACCTTCCCAAA  CGACTGATTTGTCCTGCT  GAATGGATTTACCCCCTC | 263bp  188bp  278bp  217bp  267bp  227bp  194bp  243bp  171bp |

**Table S2 The reference genome alignment statistics**

| Sample | Clean reads | Clean bases | % ≥ Q20 | Uniquely mapped ratio |
| --- | --- | --- | --- | --- |
| A1  A2  A3  B1  B2  B3  C1  C2  C3  D1  D2  D3  E1  E2  E3  F1  F2  F3 | 44997958  44597394  42967308  40965884  42904658  44240812  43209104  46537872  45234344  49685022  40462564  48369704  43476768  45015140  47109022  42896152  43162510  42383820 | 6749693700  6689609100  6445096200  6144882600  6435698700  6636121800  6481365600  6980680800  6785151600  7452753300  6069384600  7255455600  6521515200  6752271000  7066353300  6434422800  6474376500  6357573000 | 96.92%  96.74%  96.8%  96.81%  97.07%  96.92%  96.58%  96.83%  96.75%  97.1%  97.05%  96.93%  96.77%  96.66%  96.67%  96.7%  96.57%  96.5% | 88.47%  88.31%  87.98%  90.71%  90.44%  90.11%  89.59%  90.48%  88.64%  89.49%  90.23%  90.46%  95.59%  95.86%  93.77%  95.93%  95.34%  96.06% |

A: female mule ducks, B: male mule ducks, C: female Jinding ducks, D: male Jinding ducks, E: female Muscovy ducks, F: male Muscovy ducks.

**Table S3 Protein-Protein interaction table**

| Sample | Node1 | Node2 | Score |
| --- | --- | --- | --- |
| A_vs_B  C_vs_D  E_vs_F  A_vs_E  A_vs_C  B_vs_D  B_vs_F | BMPR2  IGFBP5  GABRG2  CYP19A1  PENK  SERPING1  GABRG2  BMP2  ESR1  IHH  GABRG2  MRPL18  NDUFB4  EGF  MRPL17  SERPING1  DBH  POMC  AGXT  APOB | BMPR1B  BMP2  BMP7  IGF−I  VEGFA  PAPPA  GABRB2  GABRA1  GABRA3  GABRA2  HSD17B1  PNOC  C1S  GABRB2  GABRA1  GABRA5  GABRA3  GABRA2  BMPR1B  BMPR2  CHRD  RUNX2  NR5A2  CYP19A1  GREB1  NR5A2  BOC  CDON  PTCH1  DISP2  GABRB2  GABRA1  GABRA4  MRPL9  MRPL42  MRPL58  MRPL51  MRPL38  NDUFS6  NRG1  BTC  EREG  VWF  MRPL9  MRPS36  MRPL51  C1QC  C1QA  C1S  DDC  TH  PNMT  SST  GAL  CRHR2  MC5R  PCSK1  DRD2  HAO2  EHHADH  HAO1  GPT2  AGXT2  APOA1  SERPINC1  SCARB1 | 0.95  0.964  0.972  0.998  0.989  0.951  0.984  0.986  0.968  0.969  0.981  0.96  0.989  0.984  0.986  0.986  0.968  0.969  0.976  0.964  0.984  0.955  0.951  0.966  0.961  0.951  0.969  0.96  0.998  0.956  0.984  0.986  0.968  0.991  0.958  0.994  0.967  0.971  0.996  0.976  0.972  0.973  0.971  0.997  0.964  0.969  0.965  0.968  0.989  0.989  0.97  0.99  0.971  0.951  0.953  0.987  0.951  0.951  0.996  0.961  0.993  0.953  0.985  0.997  0.979  0.971 |

Note: node1 and node2 are interacting genes.
